# The Cx43-like Connexin Protein Cx40.8 Is Differentially Localized during Fin Ontogeny and Fin Regeneration

**DOI:** 10.1371/journal.pone.0031364

**Published:** 2012-02-08

**Authors:** Sarah V. Gerhart, Diane M. Eble, R. Michael Burger, Stefan N. Oline, Ana Vacaru, Kirsten C. Sadler, Rebecca Jefferis, M. Kathryn Iovine

**Affiliations:** 1 Department of Biological Sciences, Lehigh University, Bethlehem, Pennsylvania, United States of America; 2 Department of Medicine-Division of Liver Disease, Department of Regenerative and Developmental Biology, Mount Sinai School of Medicine, New York, New York, United States of America; University of Birmingham, United Kingdom

## Abstract

Connexins (Cx) are the subunits of gap junctions, membraneous protein channels that permit the exchange of small molecules between adjacent cells. Cx43 is required for cell proliferation in the zebrafish caudal fin. Previously, we found that a Cx43-like connexin, *cx40.8*, is co-expressed with *cx43* in the population of proliferating cells during fin regeneration. Here we demonstrate that Cx40.8 exhibits novel differential subcellular localization *in vivo*, depending on the growth status of the fin. During fin ontogeny, Cx40.8 is found at the plasma membrane, but Cx40.8 is retained in the Golgi apparatus during regeneration. We next identified a 30 amino acid domain of Cx40.8 responsible for its dynamic localization. One possible explanation for the differential localization is that Cx40.8 contributes to the regulation of Cx43 *in vivo*, perhaps modifying channel activity during ontogenetic growth. However, we find that the voltage-gating properties of Cx40.8 are similar to Cx43. Together our findings reveal that Cx40.8 exhibits differential subcellular localization *in vivo*, dependent on a discrete domain in its carboxy terminus. We suggest that the dynamic localization of Cx40.8 differentially influences Cx43-dependent cell proliferation during ontogeny and regeneration.

## Introduction

Connexins, the subunits of gap junction channels, are part of a large multigene family that includes about 20 genes in mammals [1]. Connexins are integral membrane proteins that consist of four transmembrane domains, two extracellular loops, one cytoplasmic loop, and cytoplasmic amino- and carboxy- termini. Six connexin proteins oligomerize to form one connexon (or hemichannel), and the docking of two connexons at the plasma membranes of neighboring cells forms a single gap junction channel. Channels associate together at the plasma membrane to form gap junction plaques, permitting the passage of ions and small molecules (<1200 Da) between adjacent cells. Interestingly, most tissues express a unique complement of 2–7 connexin genes, suggesting that distinct homomeric and heteromeric gap junction channels contribute to cell-cell coupling and functional diversity (reviewed in [2]). Channel composition can determine metabolite selectivity and specificity [3], [4], [5]. One possible role of this diversity in connexin expression is that regulated hetero-oligomerization of different isotypes may in turn regulate tissue function.

Gap junctional intercellular communication (GJIC) is necessary for normal tissue development, evidenced by the identification of mutations in connexins that cause human disease [2], [6], [7]. In particular, missense mutations in human *CX43* cause the craniofacial and limb skeletal malformations associated with oculodentodigital dysplasia (ODDD) [8]. Importantly, the function of Cx43 appears to be conserved during skeletal morphogenesis. Mutations in zebrafish *cx43* cause short bony fin ray segments associated with the *short fin* (*sof*) phenotype [9], [10]. In addition to the *sof ^b123^* allele that has reduced mRNA and protein levels [11], [12], three non-complementing ENU-induced alleles were shown to cause missense mutations (*sof ^j7e1^* codes Cx43-F30V, *sof ^j7e2^* codes Cx43-P191S, and *sof ^j7e3^* codes Cx43-F209I). Each missense allele can form gap junction plaques, but channels exhibit aberrant ionic coupling properties [10]. Recent studies revealed a positive correlation between segment length, level of cell proliferation, and level of GJIC [10], [12]. Therefore, the zebrafish fin represents a valuable system to understand how Cx43 function contributes to the development of the vertebrate skeleton, including cell proliferation and bone growth.

The zebrafish caudal fin consists of 16–18 segmented bony rays, where fin length depends on the number and size of bony segments [13]. In addition to ontogenetic growth, fins also have the capacity for regenerative growth, where lost tissue is replaced rapidly due to an accelerated growth rate. Amputation is immediately followed by wound healing and establishment of the regeneration blastema (reviewed in [14], [15]). The blastema is a specialized structure composed primarily of proliferating cells, and is required for outgrowth. Interestingly, expression of *cx43* is up-regulated in the proliferating cells of the blastema [9], and all four *sof* alleles exhibit reduced levels of cell proliferation during regeneration [12]. Moreover, *cx43*-gene knockdown also causes reduced levels of cell proliferation [12]. Thus, Cx43 is required to promote cell proliferation during the rapid growth of fin regeneration. It remains unclear how Cx43 activity is regulated in this tissue. One possibility is that other connexins accomplish this role.

The zebrafish genome includes a large connexin gene family (n = 37) [16], almost twice the size of human (n = 21) and mouse (n = 20) families. Previously, we identified and characterized *cx40.8*, a *cx43*-like gene in the zebrafish genome [17]. The *cx40.8* gene shares 80% nucleotide identity with *cx43*, and is co-expressed with *cx43* in the population of dividing cells during fin regeneration. Therefore Cx40.8 is a reasonable candidate for influencing Cx43 function. However, Cx40.8-EGFP localized to intracellular vesicles and not to gap junction plaques when expressed alone in HeLa cells [17], in contrast to typical connexins such as zebrafish Cx43-EGFP [10]. Although, co-transfection with Cx43-mApple permits Cx40.8-EGFP to localize to the plasma membrane in gap junction plaques. Since *cx43* and *cx40.8* are both expressed in the proliferating cells of the regenerating fin, and since Cx43 and Cx40.8 can co-localize to common gap junction plaques in HeLa cells, one possibility is that Cx40.8 influences Cx43 function in dividing cells. Here, we test this hypothesis during zebrafish fin regeneration. Strikingly, we find that Cx40.8 exhibits differential subcellular localization either to the plasma membrane (during ontogeny) or to the Golgi apparatus (during regeneration). This dynamic localization is dependent on a 30 amino acid sequence immediately following the fourth transmembrane-spanning domain (TM4) of Cx40.8. We also show that that the channel properties of Cx40.8 are similar to Cx43, suggesting that Cx40.8 does not directly influence Cx43-based GJIC. Together, these findings reveal that Cx40.8-dependent subcellular localization is correlated with the growth rate of fins, perhaps by regulating a non-channel function of Cx43.

## Results

### Cx40.8 is differentially localized during ontogeny and regeneration

**Figure 1:**
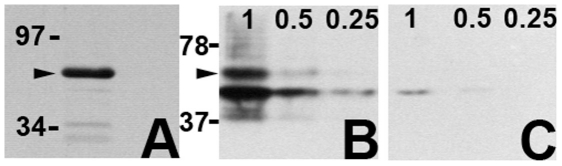
The Cx40.8 antibody is specific. (A) An antibody raised against a Cx40.8 carboxy-terminal peptide detects a single major band from regenerating fin lysates (arrowhead). Non-competed (B) and competed (C) anti-Cx40.8 antibody was used to detect bacterially expressed GST-Cx40.8CT. Decreasing volumes of a concentrated lysate were loaded in each lane on two identical gels. When the antibody was pre-incubated with the Cx40.8 target sequence (i.e. competed), antibody binding was reduced in all lanes compared with non-competed antibody. Arrowhead points to the full length product. Bands at molecular weights smaller than the full-length fusion protein represent degradation products.

Previous studies have shown that Cx40.8 is restricted to intracellular vesicles when expressed in HeLa cells [17]. To evaluate Cx40.8 localization *in vivo*, an antibody was generated against an internal peptide located in the carboxy terminus (see Materials and Methods). First we evaluated antibody-specificity using lysates prepared from regenerating fins. A single band was identified at the expected size (Figure 1A), demonstrating that the antibody recognizes Cx40.8 in fins. Peptide competition experiments confirmed the specificity of the antibody. Antibody recognition for its epitope was challenged by prior incubation with the peptide used to generate the antibody (i.e. “competed”). We prepared lysates from bacteria expressing a GST-Cx40.8CT fusion protein, and loaded increasing amounts of lysate on two identical SDS gels, followed by immunoblotting. One blot was probed with the anti-Cx40.8 antibody and the other was probed with the “competed” antibody. As expected, the immunoblot treated with competed antibody showed reduced antibody binding to the GST-Cx40.8CT fusion protein compared to the non-competed antibody (Figure 1B,C). Together, these results demonstrate that the anti-Cx40.8 antibody is specific and recognizes endogenous Cx40.8 protein by immunoblotting.

**Figure 2:**
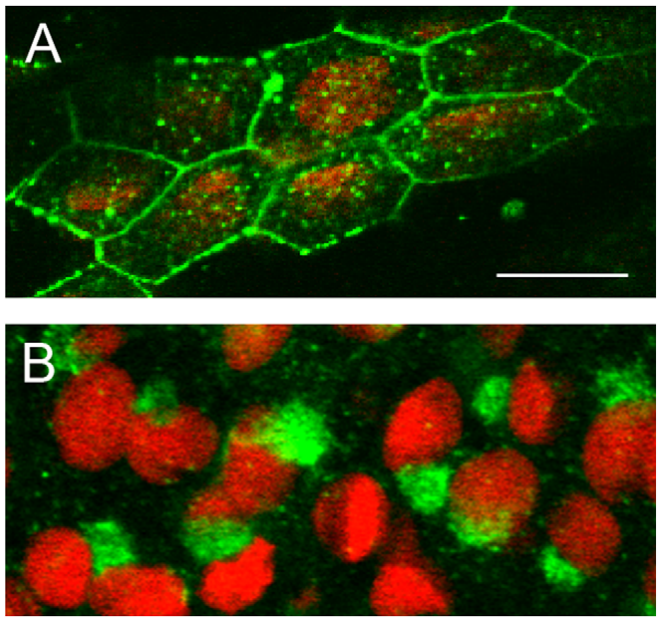
Cx40.8 is localized to different subcellular compartments during ontogeny and regeneration. (A) During ontogeny, Cx40.8 immunofluorescence (green) on whole fins counterstained with propidium iodide (red) shows that Cx40.8 locates to the plasma membrane and vesicles. (B) During regeneration, Cx40.8 is intracellular and staining is consistent with the Golgi apparatus.

Next, Cx40.8 localization in fins was evaluated during ontogenetic and regenerative growth by confocal microscopy (Figure 2). During fin regeneration, Cx40.8 is found intracellularly in crescent-like structures adjacent to the nuclei, consistent with localization in the Golgi apparatus (Figure 2B). These results were consistent with previous findings that Cx40.8-EGFP remains in intracellular vesicles in HeLa cells, and were therefore not unexpected. In contrast, during ontogeny, Cx40.8 is observed at the plasma membrane (Figure 2A), suggesting a dynamic localization of Cx40.8 that depends on growth status of the fin. Importantly, Cx43 was not found to exhibit differential localization, but is located at the plasma membrane during both ontogeny and regeneration [12]. Thus, given that the Cx43 and Cx40.8 antibodies exhibit distinct immunolocalization patterns in regenerating fins, the newly developed Cx40.8 antibody does not cross-react with endogenous Cx43. Our findings described above reveal that during the relatively slow growth of ontogeny, Cx40.8 is located at the plasma membrane. During the relatively rapid growth of regeneration, Cx40.8 is located intracellularly. Together with our previous findings that *cx43* and *cx40.8* are co-expressed in the population of dividing cells [17], and that Cx43 is required for cell proliferation [12], it is possible that Cx40.8 localization to the plasma membrane attenuates Cx43-dependent cell proliferation during ontogeny.

**Figure 3:**
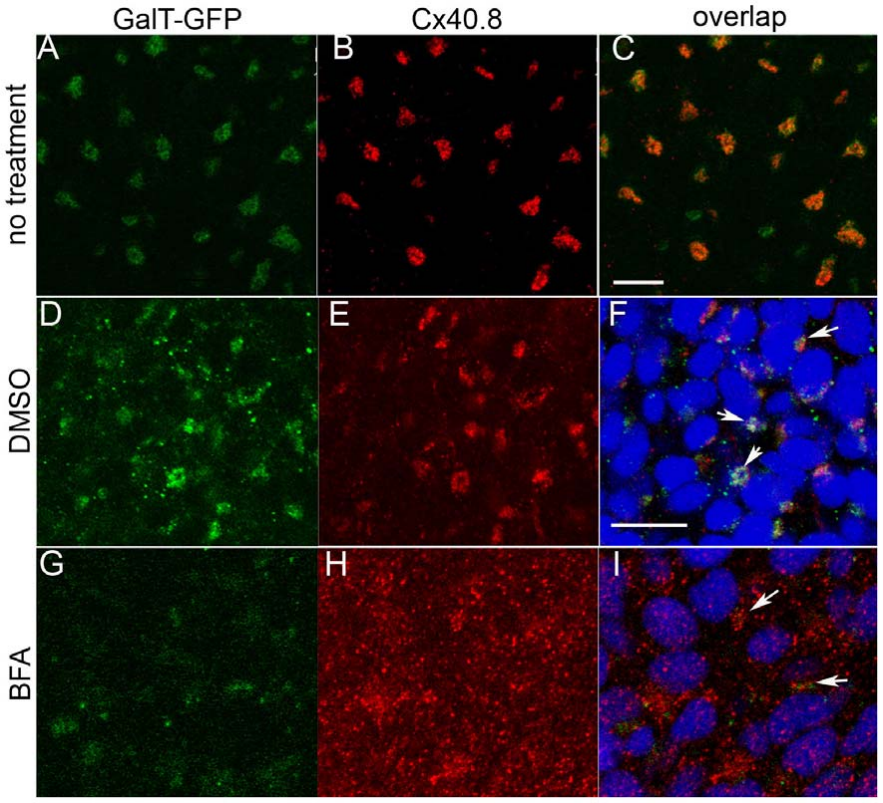
Cx40.8 co-localizes with the GalT-GFP transgene found in the Golgi during regeneration. (A–C) Co-localization in untreated regenerating fins. (D–F). Co-localization in regenerating fins treated with 0.1% DMSO carrier. (G–I) Fins injected with10 µg/ml BFA/0.1% BFA to disrupt the Golgi show dispersal of both Cx40.8 and GalT-GFP signals. In D-I, nuclei (blue) are stained with TO-PRO3 detected in the far red channel. Arrows point to areas of overlap in F (intact Golgi) and I (remnants of intact Golgi). Scale bars in C and F, 10 µm.

We next directly assessed whether Cx40.8 is localized to the Golgi apparatus during fin regeneration. To accomplish this, we utilized a novel zebrafish transgenic line that tags the amino-terminal domain of the Golgi-retained enzyme galactotransferase (GalT) with GFP (i.e. *Tg*(*bact*:*galT-gfp*)). In the transgenic line, GalT-GFP is expressed under the control of the zebrafish β-actin promoter (AV and KCS, in preparation), resulting in GFP-labeled Golgi apparatus in most cells of the fin. We found that Cx40.8 immunostaining co-localizes with GalT-GFP (Figure 3 A–C). To further establish that the two labels co-reside in the Golgi apparatus, we treated regenerating fins with a drug that specifically disrupts the Golgi apparatus, Brefeldin A (BFA, [18]). BFA should cause dispersal of both Cx40.8 and GalT-GFP if they are in fact Golgi residents. In fin rays injected with the carrier DMSO, GalT-GFP and Cx40.8 co-localized as expected (Figure 3 D–F). In contrast, fin rays injected with 10 µg/ml BFA exhibited disruption of both the Cx40.8 signal and the GalT-GFP (Figure 3 G–I). Together, these data provide compelling evidence that newly synthesized Cx40.8 localizes to the Golgi apparatus during fin regeneration. We speculate that it is eventually mobilized to the plasma membrane following the transition from regenerative to ontogenetic fin growth. In order to reveal how Cx40.8 may be retained in the Golgi, we next attempted to define the region of Cx40.8 responsible for its intracellular location.

### A carboxy-terminal domain downstream of TM4 is responsible for the intracellular localization of Cx40.8

**Figure 4:**
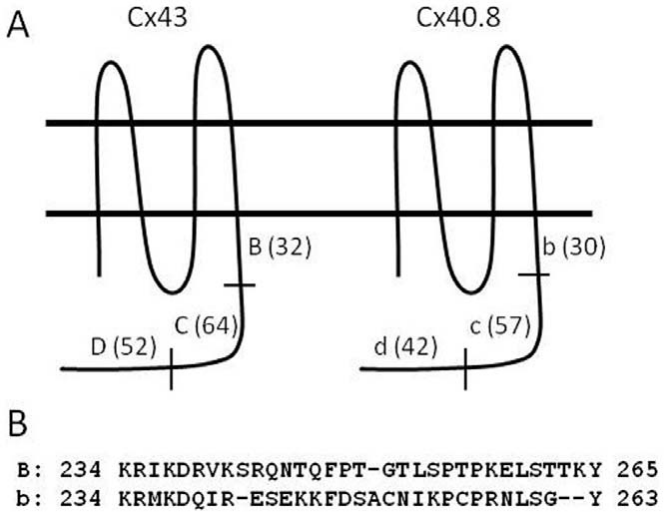
Cartoon illustrating different domains of Cx43 and Cx40.8 used to generate protein chimeras. (A) Full length sequences of Cx43 and Cx40.8. The amino terminus through the end of the fourth transmembrane-spanning domain was not modified. Swaps of the entire carboxy termini were generated, as well as swaps between domains B/b, C/c, and D/d. Uppercase refers to the Cx43 domains; lowercase refers to the Cx40.8 domains. (B) Amino acid sequence of Cx43-B vs. Cx40.8-b domains.

Since Cx40.8 is found in intracellular vesicles in HeLa cells but at the plasma membrane when co-transfected with Cx43 [17], we reasoned that Cx40.8 may contain an intrinsic signal regulating its subcellular localization. To test this hypothesis, multiple chimeric forms of Cx43 and Cx40.8 were generated to identify the domain responsible for the intracellular localization of Cx40.8. These chimeras were generated as EGFP fusion proteins in order to directly visualize subcellular localization. Cx43 and Cx40.8 are about 80% identical at the amino acid levels, where the sequences of the carboxy-termini are the least conserved [17]. Therefore, we subdivided the carboxy termini of Cx43 and Cx40.8 into three similarly sized regions, BCD for Cx43 and bcd for the analogous regions in Cx40.8 (Figure 4).

**Figure 5:**
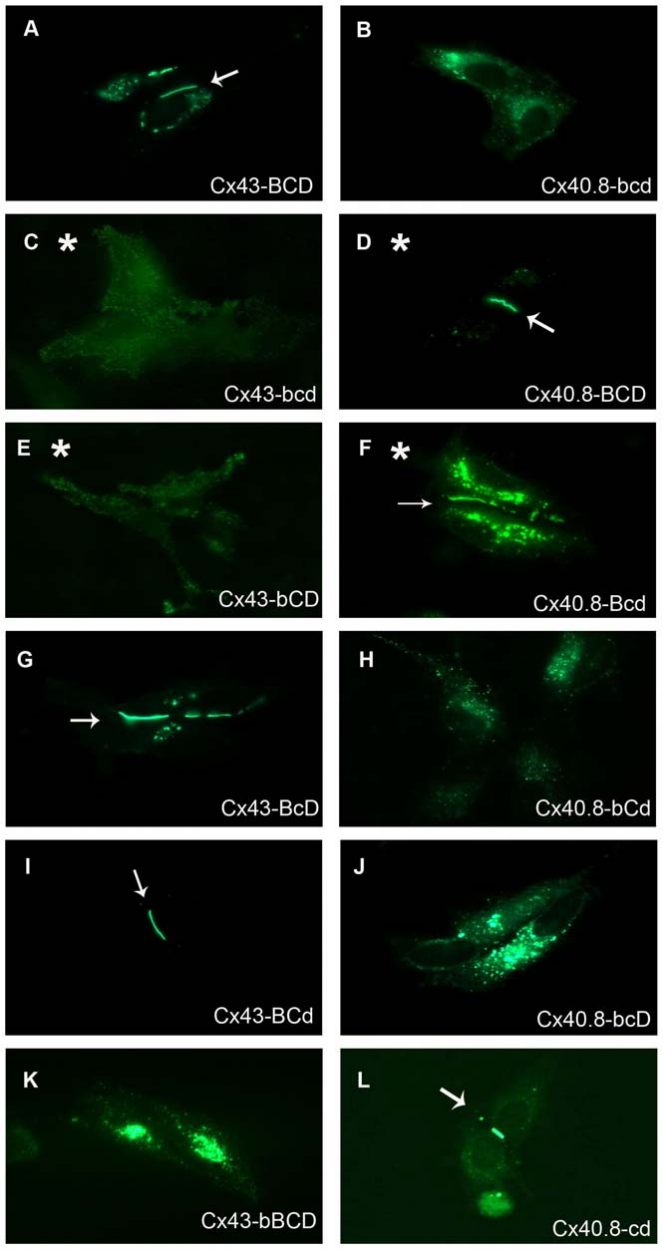
The Cx40.8-b domain is responsible for the intracellular localization of Cx40.8. HeLa cells were singly transfected with EGFP fusions of each construct. (A) Cx43-BCD-EGFP, (B) Cx40.8-bcd-EGFP, (C) Cx43-bcd-EGFP, (D) Cx40.8-BCD-EGFP, (E) Cx43-bCD-EGFP, (F) Cx40.8-Bcd-EGFP, (G) Cx43-BcD-EGFP, (H) Cx40.8-bCd-EGFP, (I) Cx43-BCd-EGFP, (J) Cx40.8-bcD-EGFP, (K) Cx43-bBCD-EGFP, (L) Cx40.8-cd-EGFP. Asterisks denote constructs in which the localization depends on a component of the carboxy terminus. Arrows identify gap junction plaques.

To test whether the carboxy terminus is indeed responsible for subcellular location, we first generated chimeras in which the carboxy termini of Cx43 and Cx40.8 were swapped (i.e. Cx43-bcd-EGFP contains the N-terminus of Cx43 but the C-terminus of Cx40.8 and Cx40.8-BCD-EGFP contains the N-terminus of Cx40.8 but the C-terminus of Cx43). Results show that Cx40.8-BCD-EGFP forms gap junction plaques at the plasma membrane, resembling the behavior of Cx43, whereas Cx43-bcd-EGFP remains intracellular, resembling the behavior of Cx40.8 (Figure 5C,D). Thus, the putative signal responsible for connexin localization appears to reside in the C-termini of these connexins. To determine more precisely the Cx40.8 localization domain, chimeras were generated between the three different sub-domains. Importantly, swapping the B/b domains had the same effect on connexin location as swapping the entire carboxy-termini suggesting that this domain is responsible for subcellular localization. Indeed, the effect was reciprocal, such that Cx43-bCD-EGFP was located in intracellular vesicles, while Cx40.8-Bcd-EGFP was found at the plasma membrane in gap junction plaques (Figure 5E,F). In contrast, swapping the C/c or D/d domains did not coincidently reverse the location of the connexins (Figure 5G–J). Together, these findings demonstrate that the carboxy-terminal domain closest to TM4 (i.e. juxta-TM4) determines the subcellular localization of both Cx43 and Cx40.8. A comparison of the sequences of these domains is included in Figure 4.

To establish conclusively that the Cx40.8 “b” domain is responsible for the intracellular localization of Cx40.8 in HeLa cells, two additional constructs were tested. The first construct deletes the “b” region from Cx40.8 (i.e. Cx40.8-cd-EGFP) and results in localization of Cx40.8-cd-EGFP into gap junction plaques at the plasma membrane (Figure 5L). Thus, “b” is necessary for the restriction of Cx40.8 to intracellular vesicles. The second construct, Cx43-bBCD-EGFP, inserts the “b” region of Cx40.8 ahead of the juxta-TM4 region of Cx43. As anticipated, this construct is retained in intracellular vesicles (Figure 5K). However, an alternate “b”-containing construct, Cx43-BbCD-EGFP, allows Cx43 to travel to the plasma membrane (not shown). We conclude from these experiments that the Cx40.8 “b” domain is required for intracellular localization of Cx40.8, and that the location of the “b” sequence in the carboxy terminus contributes to its effects on connexin localization. Indeed, the juxta-TM4 domains of both Cx43 and Cx40.8 appear to directly influence the subcellular localization of each protein (i.e. provided that the domains are located immediately downstream of TM4).

**Figure 6:**
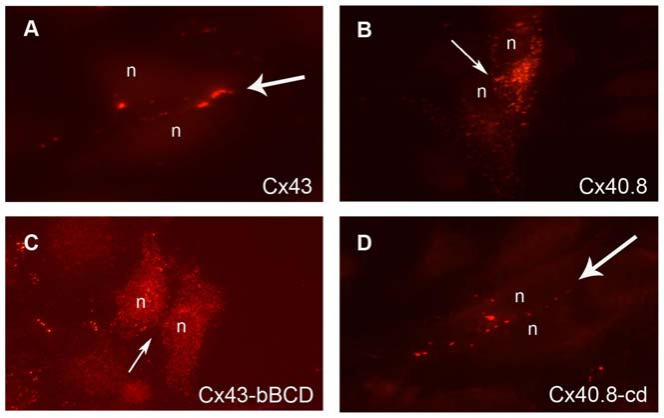
Untagged Cx43 and Cx40.8 constructs behave similarly as GFP tagged constructs in transiently transfected HeLa cells. (A) Cx43 localizes to the plasma membrane. (B) Cx40.8 is retained intracellularly. (C) Cx43-bBCD is retained intracellularly. (D) Cx40.8-cd localizes to the plasma membrane. The Cx43 antibody [12] was used to detect untagged Cx43 and untagged Cx43-bBCD. The Cx40.8 antibody was used to detect untagged Cx40.8 and untagged Cx40.8-cd. Flared arrows identify gap junction plaques located at the plasma membrane; Plain arrows identify the plasma membrane in the absence of gap junction plaques; n, nucleus.

Although EGFP fusions to connexins are widely utilized for following subcellular localization, it remained possible that such fusions could lead to aberrant localization. For example, Cx43-EGFP fusions fail to bind to the recycling factor ZO-1, leading to reduced turnover and expanded gap junctions [19], [20]. Other fluorescent proteins have been found to alter the typical trafficking of connexins (i.e. DsRed forms an obligate tetramer, causing connexins to be improperly retained, [21]). In order to rule out the possibility that the EGFP tag is confounding our results, we next evaluated untagged versions of selected constructs using connexin-specific antibodies. First, we find that untagged Cx43 and untagged Cx40.8 behave as their EGFP-tagged counterparts (Figure 6 A,B). More importantly, we find that the untagged Cx43-bBCD is retained intracellularly, confirming that “b” can impart the intracellular retention of Cx43 when inserted adjacent to TM4 (Figure 6C). Further, we find that the untagged Cx40.8-cd is able to travel to the plasma membrane and establish gap junction plaques (Figure 6D). These data indicate that the EGFP tag does not influence the trafficking of these proteins. More importantly, these data further confirm that the Cx40.8 “b” directs the intracellular retention of Cx40.8.

### Cx40.8 co-assembles with Cx43 in gap junction channels

**Figure 7:**
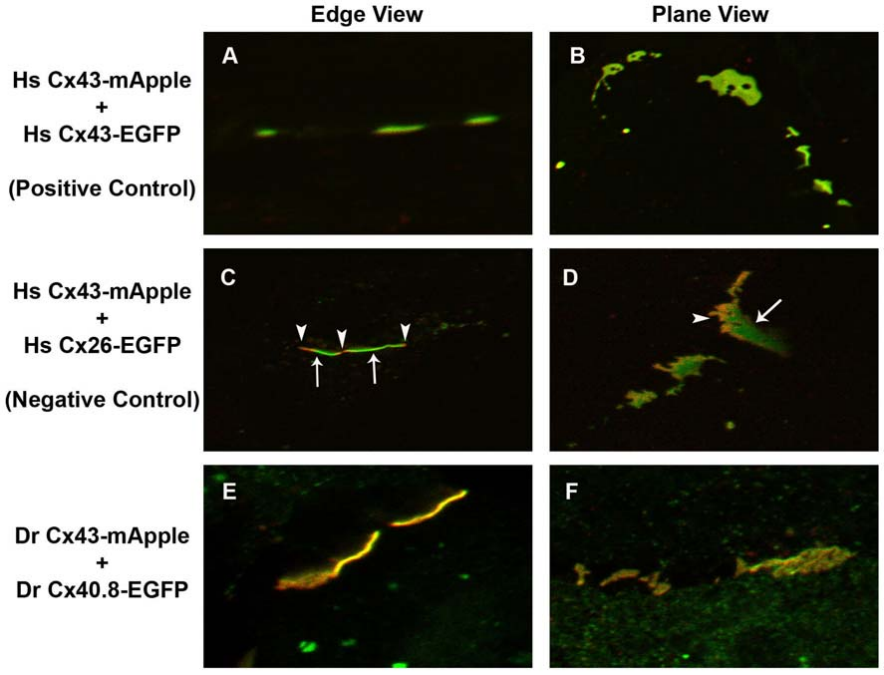
Cx43-mApple and Cx40.8-EGFP co-assemble in common gap junction channels. High resolution fluorescence microscopy was used to provide evidence for co-association of Cx43-mApple and Cx40.8-EGFP in common gap junction channels. Constructs that were co-transfected in HeLa cells are indicated to the left of the panels (A, B) *Homo sapiens* (Hs) Cx43-mApple + Hs Cx43-EGFP show uniformly yellow plaques, suggesting co-asociation. (C, D) Hs Cx43-mApple + Hs Cx26-EGFP show discrete green and red domains, revealing a lack of co-association. Arrows indicate green Hs Cx26-EGFP localization and arrowheads indicate red Hs Cx43-mApple localization. (E, F) *Danio rerio* (Dr) Cx43-mApple + Dr Cx40.8-EGFP show uniform yellow distribution.

We previously found that Cx40.8 is capable of co-localizing to gap junction plaques in HeLa cells when co-transfected with Cx43 [17], suggesting that Cx43 and Cx40.8 could associate in common gap junction channels. Increasing the primary magnification during fluorescence microscopy can be used to further investigate this hypothesis [22]. For example, co-transfection of HeLa cells with human Cx26-EGFP and human Cx43-mApple demonstrates that these connexins occupy a common gap junction plaque (Figure 7C,D and [22]). However, discrete domains of green and red are observed, revealing that Cx26-EGFP and Cx43-mApple do not hetero-oligomerize in common connexons, nor do they establish homomeric heterotypic gap junction channels (i.e. homomeric heterotypic channels would be comprised of one Cx26-EGFP connexon and one Cx43-mApple connexon). In contrast, co-transfection of human Cx43-EGFP and human Cx43-mApple results in plaques that are uniformly yellow, suggesting that the two connexins are located in common gap junction channels (Figure 7A,B). Similarly, when zebrafish Cx43-mApple was co-expressed with zebrafish Cx40.8-EGFP, the resulting gap junction plaques were yellow (Figure 7E,F). This finding suggests that Cx43 and Cx40.8 co-assemble into common gap junction channels when co-expressed in HeLa cells (see Figure S1 for single channel images).

**Figure 8:**
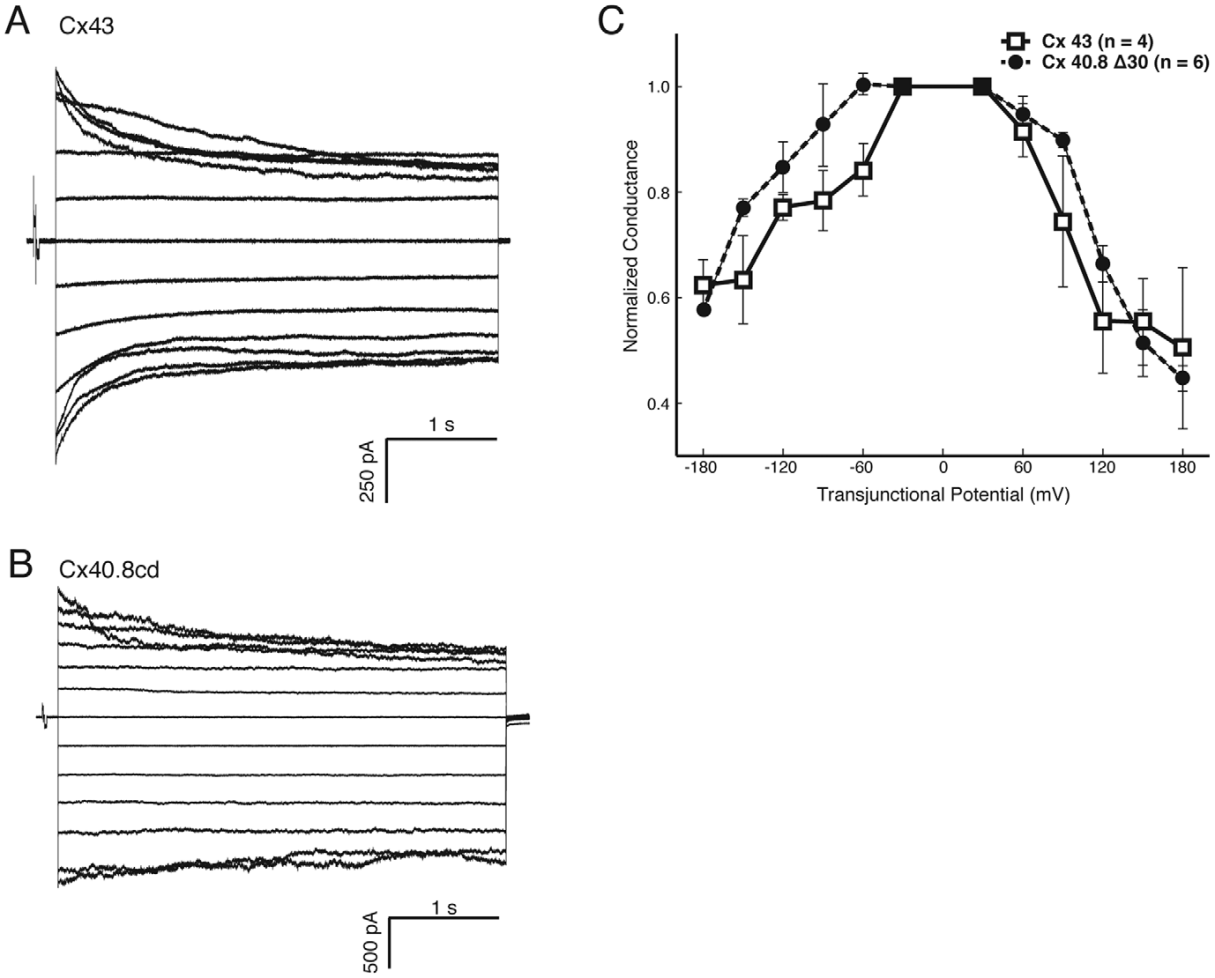
Voltage dependent conductances are similar between Cx43 and Cx40.8-cd in transiently transfected N2a cells. (A) Transjunctional current family from a Cx43 expressing cell pair. V_j_ was varied ±180 mV in 30 mV steps. (B) Currents from a Cx40.8 expressing pair. (C) Normalized steady state G_j_ measures as a function of V_j_ show similar inactivation properties between the two constructs. Error bars are SEM.

Co-association of Cx43 and Cx40.8 in common gap junction channels may cause novel functional properties, such as modulation of channel permeability or electrical-gating properties. We addressed this possibility by assessing the channel properties of Cx40.8-cd-EGFP alone, which is capable of trafficking to the plasma membrane and establishing gap junction plaques (compared to full length Cx40.8 which does not). We performed dual whole cell voltage clamp in N2a cells expressing either Cx43-EGFP or Cx40.8-cd-EGFP. Transjunctional current (I_j_) was measured while transjunctional voltage (V_j_) was varied up to ±180 mV in 30 mV steps. Voltage was stepped in one cell of the pair while the other cell remained clamped at 0 mV. Step duration was either 400 ms or 4 s. Representative current families from the 4 s protocol for cell pairs expressing Cx43-EGFP and Cx40.8-cd-EGFP constructs are shown (Figure 8 A,B). Cx43-EGFP-expressing pairs showed voltage dependent inactivation over the course of the 4 s voltage step, with transjunctional conductance (G_j_) declining to 50.4±21.5% at Vj of +180 mV (n = 4), confirming results obtained previously from paired oocyte recordings [10]. Similarly, Cx40.8-cd-EGFP containing plaques inactivated to 44.6±5.6% (n = 6). Data for the 400 ms protocols followed this trend (Cx43: 74.1±14.0%, n = 7; Cx40.8-cd: 78.5±17.2%, n = 9). Steady state G_j_ from the 4 s protocol was plotted as a function of V_j_, and normalized to the conductance at ±30 mV (Figure 8C). We find that the steady state G_j_ was similar over most of the voltage range for both constructs. These results, taken together with the high sequence homology of the two connexins [17], reveal that voltage-dependent conductance properties of Cx43 and Cx40.8 are comparable. We cannot rule out the possibility that removal of the “b” domain influences the voltage-dependent conductance of Cx40.8. However, given that the domains predicted to regulate voltage-dependent channel activity (reviewed in [23]) are highly similar between Cx40.8 and Cx43, the finding that their voltage properties are also similar is not unexpected. We therefore conclude that it is unlikely that Cx40.8 dramatically modulates the electrical properties of Cx43 channels. Other possibilities for how Cx40.8 may regulate Cx43 function will be evaluated in future experiments.

## Discussion

The four major findings of this study elucidate a novel mechanism that may regulate gap junction assembly and function. First, Cx40.8 exhibits dynamic subcellular localization in zebrafish fins, where it is retained in the Golgi apparatus during regeneration and is later mobilized to the plasma membrane during ontogeny. Thus, Cx40.8 retention is associated with the rapid growth during regeneration, and Cx40.8 at the plasma membrane is associated with the slower growth of ontogeny. Second, we identify a discrete 30 amino acid sequence near TM4 of the carboxy terminus of Cx40.8 that is required for its intracellular retention. Third, we provide evidence that Cx40.8 and Cx43 co-assemble in common gap junction channels when co-expressed in HeLa cells. Finally, we find that gap junctions composed of Cx40.8 exhibit similar voltage-dependent conductance properties as those composed of Cx43. This study is the first to document differential subcellular localization of a connexin protein based on growth status of a tissue. Thus, our findings provide the first evidence of growth-specific regulation of gap junctions.

Previous studies have found that the juxta-TM4 domain is required for connexin trafficking to the plasma membrane. For example, the mammalian Cx32-TM4 sequence (amino acids 207–219 of mammalian Cx32) has been shown to be required for localization to the plasma membrane [24]. Trafficking was suggested to involve binding of this domain to calmodulin [25]. Interestingly, the zebrafish Cx43-juxta-TM4 region (amino acids 228–263) is predicted to bind calmodulin, but the Cx40.8-juxta-TM4 region is not (http://calcium.uhnres.utoronto.ca/ctdb/no_flash.htm, data not shown). Also, the juxta-TM4 domain of mammalian Cx43 has been shown to bind microtubules, which may influence its localization to the plasma membrane [26]. The juxta-TM4 domain of Cx40.8 does not bind microtubules (data not shown). Rather, we find that the juxta-TM4 domain of Cx40.8 is required for its intracellular localization, negatively regulating trafficking to the plasma membrane. Indeed, we also find that the juxta-TM4 domain of Cx40.8 *can* cause Cx43 to be retained intracellularly (i.e. Cx43-bBCD). However, recall that the proximity of the juxta-TM4 domain to TM4 is also significant, since adding the “b” domain to Cx43 following its native “B” domain (i.e.Cx43-BbCD) does not appear to alter Cx43 trafficking. Perhaps this domain is uniquely situated to influence the trafficking or retention of connexins.

At least two other connexins have been found in intracellular compartments and not at the plasma membrane. For example, mouse Cx46 is retained in a compartment of the trans-Golgi network in ROS osteosarcoma cells, although the functional significance of this finding has not been revealed [27]. A second example is mouse Cx33, found primarily in endocytic vesicles in the testes, and has been shown to regulate the activity of Cx43 during spermatogenesis [28]. Cx33 appears to interact transiently with Cx43 at the plasma membrane, increasing the rate of Cx43 endocytosis and therefore reducing Cx43-based GJIC [29]. Examination of Cx46 and Cx33 did not reveal sequence similarity with Cx40.8 in the juxta-TM4 domains. However, phylogenetic analyses show that mouse Cx33 is most closely related to the Cx43 connexins, as is Cx40.8 [16]. Thus, zebrafish Cx40.8 and mouse Cx33 are both most closely related to the Cx43 orthologs. Perhaps co-expression of highly related connexins favors regulatory mechanisms based upon physical interaction and not on direct modification of channel properties. Indeed, given that the highest degree of sequence diversity between Cx40.8 and Cx43 occurs in the carboxy terminus, Cx40.8 may bring distinct regulatory proteins to the plasma membrane that can modify Cx43 function *in vivo*. This and other possibilities will be explored in future experiments.

Cx40.8 is a unique example of a connexin whose subcellular localization depends on the growth status of a tissue. Differential localization appears to be regulated by its juxta-TM4 domain. It is of interest to define the mechanism of this “switch.” For example, the Cx40.8 “b” sequence may interact with a hypothetical retention factor in the Golgi apparatus, preventing it from trafficking to the plasma membrane. Association between the retention factor and Cx40.8 may be reduced at the completion of regeneration, allowing the synthesized Cx40.8 to move to the plasma membrane. Once located at the plasma membrane, we suggest that Cx40.8 negatively influences Cx43 activity, contributing to reduced levels of cell proliferation associated with ontogenetic growth. It remains unclear if Cx40.8 forms homomeric heterotypic gap junction channels with Cx43 in vivo, and/or if Cx40.8 and Cx43 also hetero-oligomerize in common connexons that are initially retained in the Golgi. Both newly translated Cx43 and Cx40.8 must travel through the Golgi apparatus during fin regeneration. We have not identified a large pool of Golgi-retained Cx43 by immunofluorescence, however this does not preclude the possibility that a small portion of Cx43 is retained with newly assembled Cx40.8 connexons. Biochemical analyses will be required to determine the composition of the connexons and gap junction channels in vivo.

In conclusion, we find that Cx40.8 exhibits an unusual change in subcellular localization depending on the growth state of the zebrafish fin. Localization appears to depend on its juxta-TM4 domain, which may represent a common domain for the regulation of connexin trafficking (i.e. albeit via different mechanisms). Continued analyses will define the novel mechanisms of regulating both Cx40.8 protein location and Cx40.8-dependent regulation of Cx43 activity.

## Materials and Methods

### Statement on the ethical treatment of animals

This study was carried out in strict accordance with the recommendations in the Guide for the Care and Use of Laboratory Animals of the National Institutes of Health. The protocols used for this manuscript were approved by Lehigh's Institutional Animal Care and Use Committee (IACUC) (identification #88, approved 6-3-2011). Lehigh University's Animal Welfare Assurance Number is A-3877-01.

### Fish maintenance

The fish used in this study were derived from the C32 strain and were raised in a 14 light:10 dark photoperiod at 25°C [30]. *Tg(bact:galt-gfp)* zebrafish were generated using Invitrogen Gateway technology to fuse amino acids 1–6 from human *B4GALT1* with GFP under the control of the beta-actin promoter and inserted into the zebrafish genome using sites derived from the *tol2* transposon. Lines were maintained on the TAB14 background.

### Immunoblots and Cx40.8 antibody

Amino acids 269–285 (CSAPVPNLGYNLDTVDK) of zebrafish Cx40.8 were chosen as the antigen in conjunction with Quality Controlled Biochemicals (www.qcb.com). QCB completed peptide synthesis, immunization, rabbit maintenance, and affinity purifications of bleeds. *Escherichia coli* lysates expressing GST-Cx40.8CT fusion protein were grown to confluency before adding 0.3 mM IPTG to induce protein expression. Lysates were prepared using 50 µg/ml lysozyme in lysis buffer (100 mM Tris-HCl pH 7.5,50 mM NaCl, 10 mM EDTA pH 8.0, complete protease inhibitor cocktail, Roche). In addition, 2.2 N NaOH and 8% BME were added to the mixture. Total protein was precipitated using TCA and pellets were resuspended in SDS buffer. Samples were diluted in SDS buffer and decreasing volumes of sample were loaded in a total volume of 20 µl. For the antibody competition, identical gels were prepared. The anti-Cx40.8 antibody was either used directly (1∶2000) or following pre-incubation with the peptide made as the Cx40.8 antigen. Wildtype fins (5 dpa regenerates) were homogenized in homogenization buffer (5 mM Tris-HCl, 5 mM EDTA, 5 mM EGTA pH 8.0, 0.1 mM PEFABLOC, 1 mM Na3VO4, 1 mM Na_3_VO_4,_ protease inhibitor cocktail) using a 5 mm generator (Pro 200 homogenizer, ProScientific, Rockford, IL). Protein samples were separated using 12% SDS-PAGE before transfer to nitrocellulose membranes. Following transfer, blots were rinsed in 40% isopropanol, rinsed in distilled water, and then blocked in 5% milk in SuperTBST for 30 min at room temperature. Blots were then incubated with anti-Cx40.8 (1∶2000) for 1 hr at room temperature, rinsed for 40 min in TBST, and then incubated with peroxidase-conjugated goat anti-rabbit IgG (1∶250,000, pre-absorbed with fin tissue, Thermo Scientific, Rockford, IL) for 1 hr at room temperature. Following incubation, blots were rinsed in TBST for 40 min at room temperature. Using the Amersham ECL Plus Western Blotting Detection System (GE Healthcare, UK), blots were developed and exposed to X-ray film (CL-XPosure film, Thermo Scientific, Rockford, IL).

### Immunofluorescence and confocal microscopy

Wildtype fins (9–12 months for ontogenetic fins, or 5 dpa regenerates) were harvested and fixed in 2% PFA in 0.1 mM phosphate buffer (PB) for 30 min at room temperature. Fins were washed (3×10 min) in 25 mM PB and incubated in Trypsin/EDTA (Gibco) for 10 min on ice. Fins were next blocked (50 mM Tris-HCl; pH 7.4, 250 mM NaCl, 0.3% Triton-X 100, 6% goat serum) for 30 min at room temperature and incubated with Cx40.8 (1∶200) antibody overnight at 4°C. After incubation, fins were washed in 25 mM phosphate buffer (3×5 min) and incubated in goat anti-rabbit Alexa 488 (1∶200, Molecular probes) antibody and propidium iodide for 2 hr at room temperature followed by 1× PBS washes (3×10 min). For *Tg*(*bact*:*galT-gfp*), 5 dpa regenerates were harvested and processed identically except for the antibodies. For primary antibodies, the rabbit Cx40.8 antibody (1∶200) and the mouse EGFP (Clontech, 1∶100) antibodies were used together. The secondary antibodies were the goat anti-rabbit Alexa 546 and the goat anti-mouse Alexa 488. In the experiments with Brefeldin A (BioLegend, San Diego, CA), 70 nl of a 10 µg/ml solution was injected into the dorsal fin rays of the regenerating caudal fin. After 2 hours, fins were harvested and processed as above. TO-PRO3 (Molecular probes) was added at 1∶1000 with the secondary antibodies. Laser scanning confocal imaging was performed on whole fins. Images were acquired using a 40X N.A. 1.4 PlanApo DIC objective on an inverted microscope (Axiovert 200 M, Carl Zeiss, Jena, Germany) equipped with an LSM510META scan head. Argon ion (488), 543 HeNe (546), and 633 HeNe (far red) lasers were used to generate the excitation lines and multitrack sequential excitation was utilized to avoid bleed-through between fluorophores. Files were exported as TIFF files.

### Plasmid construction and transient transfection

The Cx43-EGFP, Cx43-mApple and Cx40.8-EGFP were previously described [17]. The constructs in which internal domains were swapped (i.e. Cx43-bCD, Cx40.8Bcd, Cx43-BcD, Cx40.8-bCd, Cx43-BCd, Cx40.8-bcD) were generated by Genewiz (http://www.genewiz.com/). Cx43-bcd, Cx40.8-BCD, Cx40.8-cd, Cx43-bBCD, and Cx43BbCD were all generated by a common strategy using two sequential rounds of PCR. For example, Cx43-bcd was generated as follows. Two chimeric internal oligos were generated. The first internal oligo shared 20 bases of 3′ complementarity to the Cx43-TM4 followed by 20 bases of complementarity to the start of the Cx40.8 carboxy tail. When paired with a 5′ oligo specific to the first 20 bases of the Cx43 coding sequence (and using Cx43-EGFP plasmid as template), the first half of Cx43 was amplified through the end of TM4, with the first 20 bp of Cx40.8 as an overhang. The second internal oligo had a 5′ overhang of the final 20 bases of Cx43-TM4 followed by the first 20 bases of the Cx40.8 carboxy tail. When paired with a 3′ oligo specific to the final 20 bases of Cx40.8 (but not the stop codon), and using Cx40.8-EGFP plasmid as template, the carboxy tail of Cx40.8 with the last 20 bp of Cx43-TM4 as an overhang is amplified. Together, these two products (sharing 40 bases of overlap across TM4 and the start of the carboxy tail) served as template for the next PCR reaction. Using the ‘outside’ oligos for amplification (i.e. the 5′ *cx43* oligo and the 3′ *cx40.8* oligo), a chimeric connexin of Cx43 through TM4 and the carboxy tail of Cx40.8 was generated. This final product was subcloned into pN1-EGFP and sequenced. The same strategy was used to generate Cx40.8-BCD, Cx40.8-cd, Cx43-bBCD, and Cx43BbCD. All internal oligos were HPLC-purified to insure that they were the correct length. The untagged constructs were generated by isolating the EcoRI fragments from their pN1-EGFP backbones. The EcoRI fragments were subcloned into the pIRES2 vector. Orientation was determined using a BamHI digest.

HeLa and N2a cells (ATCC) were stored at 5% CO_2_ and 37°C and grown in tissue culture dishes with minimal essential media supplemented with 10% FBS, antibiotics, and antimycotics (Invitrogen). The cells were seeded onto poly-L-lysine coverglasses, incubated overnight, then transfected with Superfect (Qiagen) for 3 hours using 2 µg of plasmid DNA and imaged 21–24 hours later. Transfections were evaluated by standard immunofluorescence on a Nikon Eclipse E80 microscope. For double transfections, 1 µg of each plasmid was transfected. Analyses were completed on a Nikon Eclipse TE2000-U using the 100× lens in combination with a 1.5× optivar to increase the primary magnification.

### Dual whole cell patch clamping

For dual whole cell voltage clamp recording, the poly-L-lysine cover glass was placed into a chamber at room temperature perfused with oxygenated extracellular solution (containing in mM: 135 NaCl, 5 KCl, 2 CsCl, 2 CaCl2, 1 MgCl2, 1 BaCl2, 5 dextrose, 5 HEPES, and 2 pyruvate, pH 7.2–7.4). N2a cells were imaged on a Nikon (EF-4) Physiostation equipped with epifluorescence and infra red differential interference contrast optics. GFP positive cell pairs expressing connexin constructs were located by fluorescence and imaged using a Hammamatsu C5700 video camera (Hammamatsu City, Japan). Cells contacting multiple GFP positive neighbors were excluded.

Patch pipettes were pulled from thick walled borosilicate glass capillary tubes (WPI 1B120F-4) using a two-stage puller (Narishige PC-10, Tokyo, Japan) to a resistance of 4–6 MΩ when back-filled with internal solution (containing in mM: 125 CsCl, 0.5 CaCl2, 10 EGTA, and 10 HEPES, pH 7.2). Patch pipettes were then brought into contact with cells using motorized micromanipulators (Model 7500, Siskiyou Instruments) and gigaohm seals were established. Whole cell configuration was achieved and cells were initially voltage clamped to 0 mV using a Multiclamp 700B amplifier (Molecular Devices, Sunnyvale, CA). Series resistance was compensated at 60–80%. The signal was filtered at 1–2 kHz and digitized at 10 kHz with a Digidata 1440 data acquisition board. Recording protocols were generated using Clampex 10.2 software (Molecular Devices, Sunnyvale, CA).

Voltage clamp methods followed [31]. Transjunctional potential (V_j_) was varied up to ±180 mV in steps of 30 mV. Step duration was either 400 ms or 4 s. Recordings were analyzed using Clampex 10.2 software. Transjunctional conductance values were calculated from mean transjuctional current (I_j_) amplitudes from a 15 ms window measured at 5 ms after pulse onset. Steady state G_j_ was computed from I_j_ measured at 5 ms before the termination of the pulse. G_j_ was maximal and generally linear at voltages <60 mV so steady state G_j_ was normalized to the conductance value calculated at ±30 mV. Data in the results text are expressed as percent of maximum G_j_ ± SD. Error bars in figures represent SE.

## Supporting Information

Figure S1 **Cx43-mApple and Cx40.8-EGFP co-assemble in common gap junction channels.** High resolution fluorescence microscopy was used to provide evidence for co-association of Cx43-mApple and Cx40.8-EGFP in common gap junction channels. Plane views are shown. Top: In HeLa cells co-transfected with Hs-Cx43-EGFP and Hs-Cx43-mApple, both green and red plaques completely overlap. Middle: In HeLa cells co-transfected with Hs-Cx26-GFP and Hs-Cx43-mApple, discrete domains of green and red plaques are observed. Bottom: In HeLa cells co-transfected with Dr-Cx40.8-EGFP and Dr-Cx43-mApple, both green and red plaques completely overlap.
(TIF)
